# Structural basis for the glycosyltransferase activity of the *Salmonella* effector SseK3

**DOI:** 10.1074/jbc.RA118.001796

**Published:** 2018-02-15

**Authors:** Diego Esposito, Regina A. Günster, Luigi Martino, Kamel El Omari, Armin Wagner, Teresa L. M. Thurston, Katrin Rittinger

**Affiliations:** From the ‡ Molecular Structure of Cell Signalling Laboratory, Francis Crick Institute, 1 Midland Road, London NW1 1AT, United Kingdom,; the § Section of Microbiology, Medical Research Council Centre for Molecular Bacteriology and Infection, Imperial College London, London SW7 2AZ, United Kingdom, and; the ¶ Diamond Light Source, Harwell Science and Innovation Campus, Chilton, Didcot OX11 0DE, United Kingdom

**Keywords:** glycosyltransferase, Salmonella enterica, protein structure, enzyme mechanism, bacterial toxin, X-ray crystallography, arginine modification, bacterial effectors, glycosyltransferase type-A, SseK3, UDP-GlcNAc, GT-A family, structural analysis

## Abstract

The *Salmonella*-secreted effector SseK3 translocates into host cells, targeting innate immune responses, including NF-κB activation. SseK3 is a glycosyltransferase that transfers an *N*-acetylglucosamine (GlcNAc) moiety onto the guanidino group of a target arginine, modulating host cell function. However, a lack of structural information has precluded elucidation of the molecular mechanisms in arginine and GlcNAc selection. We report here the crystal structure of SseK3 in its apo form and in complex with hydrolyzed UDP-GlcNAc. SseK3 possesses the typical glycosyltransferase type-A (GT-A)-family fold and the metal-coordinating D*X*D motif essential for ligand binding and enzymatic activity. Several conserved residues were essential for arginine GlcNAcylation and SseK3-mediated inhibition of NF-κB activation. Isothermal titration calorimetry revealed SseK3's preference for manganese coordination. The pattern of interactions in the substrate-bound SseK3 structure explained the selection of the primary ligand. Structural rearrangement of the C-terminal residues upon ligand binding was crucial for SseK3's catalytic activity, and NMR analysis indicated that SseK3 has limited UDP-GlcNAc hydrolysis activity. The release of free *N*-acetyl α-d-glucosamine, and the presence of the same molecule in the SseK3 active site, classified it as a retaining glycosyltransferase. A glutamate residue in the active site suggested a double-inversion mechanism for the arginine *N*-glycosylation reaction. Homology models of SseK1, SseK2, and the *Escherichia coli* orthologue NleB1 reveal differences in the surface electrostatic charge distribution, possibly accounting for their diverse activities. This first structure of a retaining GT-A arginine *N*-glycosyltransferase provides an important step toward a better understanding of this enzyme class and their roles as bacterial effectors.

## Introduction

*Salmonella enterica* is an intracellular Gram-negative pathogen for which strains of the various serovars cause many diseases in humans and animals worldwide. In immunocompetent humans, non-typhoidal *Salmonella* serovars, including *S. enterica* serovar Typhimurium, typically cause self-limiting gastroenteritis (1). However, in immunocompromised individuals, non-typhoidal *Salmonella* serovars frequently cause an invasive disease that results in significant morbidity (2).

Following invasion or phagocytic uptake into the host cell, one of the key virulence determinants is the *Salmonella* pathogenicity island 2 (SPI-2)^4^-encoded type III secretion system (T3SS), which delivers ∼28 effector proteins into the host cell (3–5). A number of *Salmonella* effectors target and modify host proteins that have a role in mediating host inflammatory responses (3, 6). Three highly related *Salmonella* effectors, SseK1, SseK2, and SseK3, orthologues of the enteropathogenic and enterohemorrhagic *Escherichia coli* T3SS effector NleB1 (7), translocate via the T3SS into the host cell (8). Similarly to NleB1 (9, 10), SseK1 and SseK3 are *N*-acetylglucosamine (GlcNAc) transferases that modify the TNFR1-associated death domain protein TRADD and inhibit activation of the pro-inflammatory transcription factor NF-κB, as well as necroptotic host cell death (8). In *in vitro* experiments, SseK2 modifies the FAS-associated protein with death domain (FADD) (11), but despite detectable translocation into host cells, SseK2 is only able to inhibit NF-κB when highly overexpressed in 293ET cells (8). This suggests that a loss either of catalytic activity or of substrate interaction occurs after delivery of physiologically relevant levels of SseK2. Whether SseK2 has unidentified functions during infection that are mediated by different targets or a different sugar modification remains unknown.

Unlike mammalian *O*-linked GlcNAcylation, where GlcNAc is attached to the oxygen of the hydroxyl group of serine and threonine residues of numerous cytosolic and nuclear proteins, including those that mediate signaling (12), GlcNAcylation mediated by NleB from pathogenic *E. coli* or SseK bacterial effectors results in the addition of GlcNAc to arginine residues with an *N*-glycosidic linkage (9, 10). This distinct modification can be recognized in mammalian cells using an antibody that does not detect *O*-linked GlcNAcylation (13). Both mammalian GlcNAcylation and bacterially mediated arginine GlcNAcylation involve the transfer of sugar from an activated uridine-diphosphate carbohydrate donor substrate, UDP-GlcNAc. NleB and SseK proteins have a conserved D*X*D motif, typical of GT-A family of glycosyltransferases, where the aspartic side chains are required for the coordination of a metal divalent cation necessary for their enzymatic activity (14).

Although, to date, there are a number of structures describing *O*-glycosyltransferases and the *O*-GlcNAcylation catalytic event, the only known structure of a bacterial arginine-specific glycosyltransferase is that of the B pattern-type inverting glycosyltransferase EarP, which modifies translation elongation factor P (EF-P) by arginine rhamnosylation (15, 16). Another study reported glucosylation of arginine by the sweet corn protein amylogenin, which mediates self-glucosylation *in vitro* (17), but there have been no follow-up reports.

Sequence analysis suggests a structure for the catalytic core of NleB1 protein similar to that of *Photorhabdus asymbiotica* tyrosine-glycosyltransferase protein toxin (18) and *Clostridium difficile* toxin A and toxin B (19, 20), which glycosylate host Rho GTPases involved in the regulation of the host cytoskeleton, and the *Legionella pneumophila* Lgt1 that targets a serine residue of eEF1A, blocking protein biosynthesis (21). As well as the conserved D*X*D motif in SseK effectors, additional amino acids are conserved, including a tyrosine and glutamic acid residue, equivalent to Tyr-219 and Glu-253 in NleB1, both required for NleB1-mediated arginine GlcNAcylation of the death domain–containing protein, FADD (22). Arginine GlcNAcylation of target proteins is not observed in uninfected host cells and is irreversible by host enzymes (23) and therefore represents a potent bacterially mediated virulence mechanism. However, in the absence of structural information, the molecular details underlying arginine and UDP-GlcNAc selection are unknown.

This study presents the crystal structures of the *N*-glycosyltransferase SseK3 in its free form and bound to UDP, GlcNAc, and manganese. SseK3 adopts the classical GT-A glycosyltransferases family fold and is able to hydrolyze UDP-GlcNAc in the absence of a protein substrate. Mutational analysis of amino acids predicted to be important for substrate binding identified several conserved residues in SseK3 that are essential for arginine GlcNAcylation of TRADD and SseK3-mediated inhibition of TNFα-induced NF-κB activation, directly correlating enzymatic activity to virulence function. Structural analysis reveals the presence of an active site glutamate residue, which is conserved only in NleB and SseK proteins and not in other structurally related glycosyltransferases and is essential for enzymatic activity. The close proximity of this residue to the reactive anomeric carbon supports a double-inversion catalytic mechanism, where an intermediate enzyme–sugar is stabilized for the nucleophilic attack of the acceptor arginine side chain of the host substrates. This is the first structure of a retaining arginine glycosyltransferase bound to a hydrolyzed form of its donor substrate, representing an important step toward the understanding of this class of effector proteins.

## Results

### A divalent cation is necessary for binding of the ligand to SseK3

Mass spectrometry analysis of bacterially expressed full-length SseK3 revealed limited proteolysis, with the protein losing its first 13 and last 2 amino acids (data not shown). For the majority of T3SS effector proteins, the N-terminal 15–25 residues, often predicted to be unstructured, are required for translocation and remain uncleaved following delivery into the host cell cytosol (24, 25). This region is frequently removed before crystallization, as in the case of the *E. coli* effector protease NleC (26), or disordered, as in the case of *E. coli* virulence factor NleE (27). We therefore prepared two forms of SseK3, 14-333 and 14-335, for subsequent structural and functional characterization.

**Figure 1.**
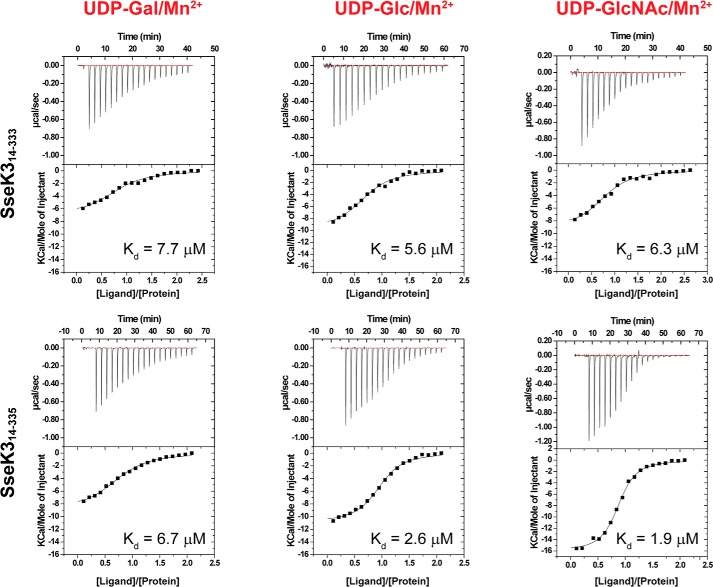
**Binding affinity of the ligand to SseK3.** Isothermal titration calorimetry curves for the interaction of SseK3(14–333) and SseK3(14–335) with UDP-galactose (*UDP-Gal*), UDP-glucose (*UDP-Glc*), and UDP-glucosamine (*UDP-GlcNAc*) are shown. The experiments were executed in the presence of 5 mm MnCl_2_. The integrated heat signals for the interaction were integrated as a function of the molar ratio of titrant to protein in the cell. The data were fitted to a 1:1 binding model, and the dissociation constants are reported for each experiment.

Glycosyltransferases require the presence of a divalent cation for activity. To test the selectivity of SseK3 for divalent cations and UDP-glucose derivatives, we compared the ability of SseK3 to interact with Mg^2+^, Mn^2+^, and three UDP-carbohydrate derivatives (UDP-GlcNAc, UDP-Glc (glucose), and UDP-Gal (galactose)) by isothermal titration calorimetry (ITC) (Fig. 1, Fig. S1, and Table 1). No binding was observed for UDP-GlcNAc to SseK3(14–335) in the absence of a coordinating metal cation (Fig. S1). When the titration experiments were performed in the presence of MgCl_2_ or MnCl_2_, the resulting binding affinity for UDP-GlcNAc in the presence of Mn^2+^ (*K_d_* = 1.9 μm) showed a 5-fold increase compared with that for Mg^2+^ (*K_d_* = 10 μm) (Fig. 1 and Fig. S1). This binding affinity is similar to that observed for *C. difficile* toxin A for UDP-Glc and manganese (*K_d_* = 11.4 μm) (28). The binding studies showed that ligand interaction is influenced by the presence of the two C-terminal residues in the longer SseK3 construct (Trp-334 and Arg-335), but both SseK3(14–333) and SseK3(14–335) bind with a lower affinity to UDP-Gal, compared with UDP-Glc and UDP-GlcNAc. UDP-GlcNAc has the highest affinity for SseK3(14–335), although it only binds marginally stronger than UDP-Glc, and manifests the largest increase in affinity due to the presence of the two C-terminal residues Trp-334 and Arg-335. Interestingly, the change in affinity appears to be associated with an increase in binding enthalpy and a loss of entropy, suggesting the occurrence of a structural reorganization upon ligand binding to SseK3 (Table 1).

**Table 1:** **Thermodynamic quantities for the interaction of SseK3 constructs and UDP-glucose derivatives**

Protein/Ligands	*K_d_*	Δ*H*	−*T*Δ*S*	Sites
	μ*m*	*kcal/mol*	*cal/mol degrees*	
SseK3(14–335)/no cation	NBD*^a^*	NBD	NBD	NBD
SseK3(14–335)/UDP-GlcNAc/Mg^2+^	10 ± 1.0	−7.4 ± 0.4	0.7 ± 0.1	0.8 ± 0.1
SseK3(14–335)/UDP-GlcNAc/Mn^2+^	1.9 ± 0.5	−15.8 ± 0.3	8.1 ± 0.2	0.9 ± 0.1
SseK3(14–335)/UDP-Gal/Mn^2+^	6.7 ± 0.8	−8.9 ± 0.3	2.0 ± 0.4	0.8 ± 0.1
SseK3(14–335)/UDP-Glu/Mn^2+^	2.6 ± 0.1	−11.4 ± 0.8	4.0 ± 0.8	0.9 ± 0.1
SseK3(14–333)/UDP-GlcNAc/Mn^2+^	6.3 ± 0.5	−8.8 ± 0.5	1.9 ± 0.6	0.8 ± 0.1
SseK3(14–333)/UDP-Gal/Mn^2+^	7.7 ± 1.6	−7.1 ± 0.4	2.6 ± 0.5	0.8 ± 0.1
SseK3(14–333)/UDP-Glu/Mn^2+^	5.6 ± 1.2	−9.8 ± 0.5	2.8 ± 0.6	0.7 ± 0.1

*^a^* NBD, no binding detected.

### Structure of SseK3

**Figure 2.**
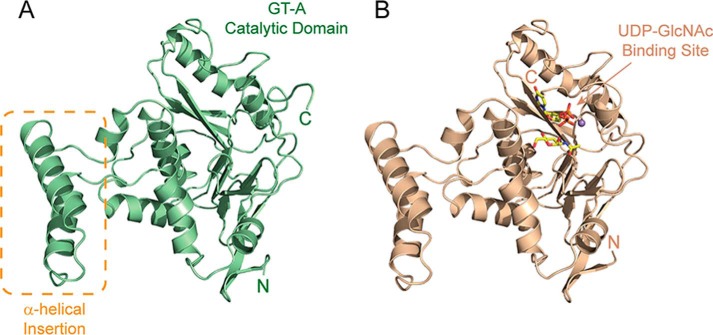
**Structure of ligand-free and bound SseK3.** Shown is a *ribbon representation* of the structure of the unbound SseK3(14–333) (*A*) and SseK3(14–335)–UDP-GlcNAc–Mn^2+^ complex (*B*). The figure shows the central GT-A catalytic domain that binds the ligand substrate and highlights the α-helical insertion that shows variability in the two protomers in the asymmetric crystallographic unit.

Crystals of SseK3(14–333) enriched with selenomethionine diffracted to good resolution, but most of them exhibited severe anisotropy, were non-isomorphous, and showed translational non-crystallographic symmetry. Attempts to solve the structure by molecular replacement using available glycosyltransferase X-ray structures or selenium SAD phasing failed. Instead, we recorded data at the I23 long-wavelength beamline at Diamond Light Source to reveal the positions of the selenium and sulfur atoms within SseK3 based on the small anomalous signals present at long wavelengths to obtain an initial model (29). This model was used as template in a molecular replacement search with a complete 2.21 Å resolution data set that did not exhibit severe diffraction anisotropy. The SseK3(14–333) crystal belongs to space group *P*2_1_2_1_2_1_ with two molecules in the asymmetric unit (a.u.) (Table 2) that overlap with a root mean square deviation (RMSD) (Cα of residues 27–328) of 0.91 Å. The structure of apo-SseK3(14–333) is reported in Fig. 2*A*. Residues 14–25 are not visible in the electronic density for either chain, nor are amino acids 329–333 of chain B and the last serine residue (Ser-333) of chain A.

**Table 2:** **Crystallographic data collection and refinement statistics**

	SseK3_long-wavelength_	SseK3_apo_	SseK3_UDP-GlcNAc_
Residues	14–333	14–333	14–335
PDB code		6EYR	6EYT
**Data collection**			
Wavelength (Å)	2.7552	0.97640	0.97950
Resolution range (Å)	49.88–2.1	58.24–2.2	48.01–2.21
Highest resolution range (Å)	2.175–2.1	2.279–2.2	2.289–2.21
Space group	P2_1_	P2_1_2_1_2_1_	P2_1_2_1_2_1_
Cell dimensions			
*a*, *b*, *c* (Å)	96.08, 75.02, 101.89	73.61, 86.05, 95.22	73.97, 85.94, 96.03
α, β, γ (°)	90, 109.52, 90	90, 90, 90	90, 90, 90
Total reflections	1,430,296 (110,092)*^a^*	230,518 (23,312)	186,687 (10,819)
Unique reflections	62,053 (4764)	31,246 (3104)	30,891 (2847)
Multiplicity	23.0 (22.3)	7.4 (7.5)	6.0 (3.8)
Completeness (%)	77.31 (59.94)	99.42 (99.52)	98.54 (91.72)
Mean *I*/σ(*I*)	13.34 (1.79)	7.79 (2.80)	6.99 (1.18)
Wilson *B*-factor	39.47	23.08	31.1
*R*_merge_	0.1655 (1.796)	0.1509 (0.6784)	0.1806 (1.034)
*R*_meas_	0.1693 (1.84)	0.1624 (0.7296)	0.1974 (1.185)
*R*_pim_	0.03484 (0.3808)	0.05927 (0.2654)	0.07853 (0.564)
CC1/2	0.997 (0.707)	0.994 (0.888)	0.995 (0.55)
CC*	0.999 (0.91)	0.999 (0.97)	0.999 (0.842)
Phasing			
Resolution cutoff (Å)	3.5		
Sites (found/expected)	40/40		

**Refinement**			
Reflections used in refinement		31,203 (3092)	30,886 (2847)
Reflections used for *R*_free_		1543 (131)	1530 (124)
*R*_work_		0.2171 (0.2755)	0.1963 (0.2676)
*R*_free_		0.2582 (0.2966)	0.2408 (0.2891)
CC (work)		0.950 (0.846)	0.959 (0.783)
CC (free)		0.918 (0.796)	0.941 (0.784)
Number of non-hydrogen atoms		5185	5311
Protein atoms		4867	4940
Ligand atoms			86
Solvent atoms		318	285
Protein residues		610	618
RMSD bond lengths (Å)		0.003	0.005
RMSD angles (degrees)		0.67	0.78
Ramachandran favored (%)		98.51	98.37
Ramachandran allowed (%)		1.49	1.63
Ramachandran outliers (%)		0	0
Rotamer outliers (%)		0	0.37
Clashscore		7.05	8.86
Average *B*-factor (Å^2^)		30.15	33.58
Macromolecules (Å^2^)		29.95	33.3
Solvent (Å^2^)		33.27	37.94
Ligands (Å^2^)			35.49

*^a^* Highest-resolution shell values are given in parentheses.

The structure of SseK3(14–335) at 2.20 Å in complex with UDP, GlcNAc, and Mn^2+^ (Fig. 2*B*) was solved by molecular replacement using the apo-structure. SseK3(14–335) crystals belong to the same space group as the shorter unbound construct, with two molecules in the asymmetric unit (Table 2). The two chains in the a.u. overlap with an RMSD in Cα positions of 0.88 Å for residues 27–335. No electronic density is visible for amino acids 14–26 for either chain in the a.u., whereas all of the C-terminal residues are observable. Residues 27–321 of SseK3 in the apo and ligand-bound forms overlap with an RMSD of 0.84 Å, whereas residues 322–332 of SseK3(14–333) are unstructured with an average B factor of 45.5 Å^2^, compared with the average macromolecule B factor of 33.3 Å^2^.

The SseK3 catalytic core domain (∼250 residues) shows the classic features of the large glycosyltransferase type-A family of enzymes (GT-A) consisting of a single module composed of a central parallel β-sheet core flanked by a number of α-helices (14). The structure contains an additional small domain, spanning residues 134–171, containing two extra α-helices that are here named the α-helical insertion (Fig. 2). This protruding region, especially the position of the residues in the loop 148–154, is differently tilted in the two SseK3 copies present in the crystallographic asymmetric unit, most likely due to non-identical crystal contacts.

Using the structure of SseK3(14–335) as template, a DALI (30) search for homologous structures yielded high scores of 17.4, 16.9, 16.7, and 16.2 for the GT-A family members lethal toxin (LT) from *C. sordelii* in complex with UDP-Glc (PDB code 2VKD) (31), *P. asymbiotica* ToxG bound to UDP-GlcNAc (PDB code 4MIX) (18), *C. difficile* toxin A bound to UDP-Glc (PDB code 3SRZ) (20), and *C. difficile* toxin B bound to UDP and Glc (PDB code 2BVL) (19), respectively. According to the CAZy (Carbohydrate-Active Enzymes) database (32), all of these enzymes are classified as sugar stereochemistry–retaining glycosyltransferases.

**Figure 3.**
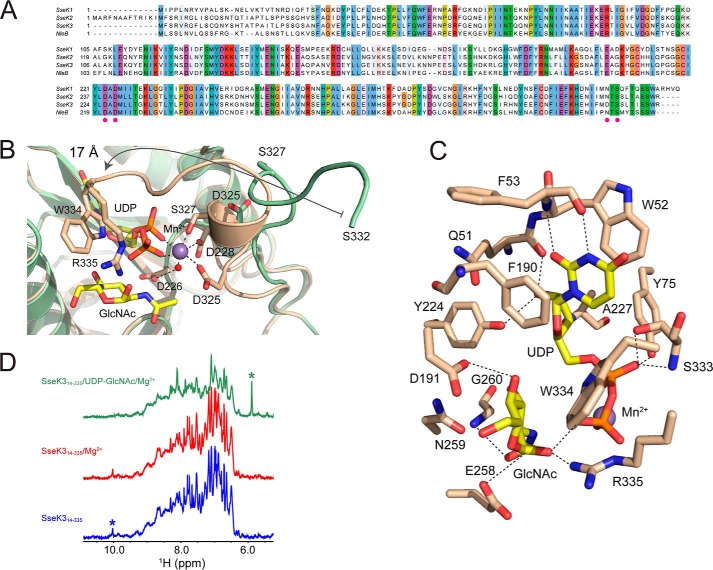
***Close-up* of the catalytic core of SseK3.** *A*, sequence alignment of the SseK effectors family and their *E. coli* ortholog NleB. The conserved residues involved in the metal coordination, including the D*X*D motif, are reported as *magenta dots* on the sequence alignment. Shown are *close-ups* of the residues that participate in the coordination of the Mn^2+^ ion (*B*) and in the ligand interaction (*C*). *D*, downfield region of the proton NMR spectra of SseK3, showing that the addition of Mg^2+^ and UDP-GlcNAc quashes the sharp resonances present in the spectrum of unbound SseK3. The *asterisks* show the resonances from a complex multiplet around 6 ppm originating from the UDP-GlcNAc (*green*) and the resonance at 10 ppm from the Hϵ1 of Trp-334 (*blue*).

Due to the length of the crystallization process and to the hydrolysis capabilities of SseK3(14–335), in the structure co-crystallized in the presence of UDP-GlcNAc and Mn^2+^, the ligand is hydrolyzed with the UDP and *N*-acetyl α-d-glucosamine still present in the active site (Fig. 2*B* and Fig. S2). A similar observation was made for *C. difficile* toxB co-crystallized in the presence of UDP-Glc (19). A comparison with the apo-structure reveals that the C-terminal amino acids in the ligand-bound SseK3 fold back onto the ligand to produce a short segment of α-helix, with the last two residues Trp-334 and Arg-335 directly interacting with the UDP and GlcNAc (Fig. 3, *B* and *C*).

To investigate the conformation of this C-terminal region in the apo state, we recorded a 1D proton NMR spectrum of apo-SseK3(14–335). The spectrum has a downfield region, encompassing resonances from the backbone and side-chain amides and aromatic side-chain aliphatic protons that show an overall chemical shift dispersion typical of a folded protein (Fig. 3*D*). The spectrum has a surprising number of sharp resonances, including a signal at 10 ppm, absent in the spectrum of SseK3(14–333) (Fig. S2*C*), probably corresponding to the side-chain Hϵ1 of the C-terminal residue Trp-334. The sharp signals in the spectrum of the free protein are broadened when UDP-GlcNAc and MgCl_2_ are added to the solution, suggesting a conformational rearrangement and loss of flexibility. Reorganization of the C terminus in the SseK3 structure upon ligand binding correlates both with the NMR downfield resonance collapse in the 1D proton spectrum and with the loss of entropy upon binding detected by the ITC experiments.

### The active site

SseK3 contains the classic GT-A D*X*D motif (^226^DAD^228^) that coordinates the divalent manganese cation in an octahedral geometry (Fig. 3, *A* and *B*). Mn^2+^ interacts with the carboxylate oxygens of Asp-228 and Asp-325, the UDP diphosphate, and the hydroxyl group of Ser-327, as well as an ordered water molecule, which bridges, via hydrogen bonds, the metal atom and the carboxyl group of Asp-226 of the D*X*D motif (Fig. 3*B*). All of the metal-coordinating residues are conserved across this effector family apart from SseK3 Asp-325, which is an asparagine residue in the other family members (Fig. 3*A*). Most C-terminal residues participate in interactions that lock the phosphate groups in the correct orientation. Ser-333, Trp-334, and Arg-335 all interact via hydrogen bonds with the oxygen atoms of the diphosphate. The last residue visible in the structure of SseK3(14–333), serine 332, translates 17 Å from its position in the apo-structure to interact directly with a β-oxygen of the UDP (Fig. 3*B*).

The aromatic portion of the ligand is held in place by a series of interactions that include hydrogen bonds of the backbone amide and carbonyl of Phe-53 with the N3 and O2 of the ring and the π-stacking of the uracil by Trp-52 and Phe-190 (Fig. 3*C*). The ribose C2 hydroxyl group interacts with both the backbone carbonyl of Gln-51 and the hydroxyl group of Tyr-224, whereas the C3 -OH group forms a hydrogen bond with the backbone amide of Ala-227.

The *N*-acetyl α-d-glucosamine in the active site is stabilized by a series of interactions that involve the anomeric C1 hydroxyl group, at a distance of 3.6 Å from the phosphorus atom to which it was bound, and the guanidine group of Arg-335, with hydrophobic interactions also established between the sugar and the aromatic ring of Trp-334 (Fig. 3*C*). The C2 acetyl group oxygen, absent in UDP-Glc, forms a hydrogen bond (3.13 Å) with the backbone amide of Gly-260. The same interaction can be seen between Gly-405 and the sugar acetyl group in the structure of the pneumococcal transferase GtfA in complex with UDP and GlcNAc (PDB code 4PQG) (33) and might contribute to the binding preference of SseK3 for UDP-GlcNAc compared with UDP-Glc. The structural position of Gly-260 is substituted by a bulky glutamine side chain in clostridial toxin A and toxin B (Fig. 3*C*), suggesting an explanation for preference of these toxins for UDP-Glc (19, 20). The GlcNAc C4 and C6 hydroxyl groups in the structure of ligand-bound SseK3 are locked in the active site by the side-chain carboxylate of Asp-191, which may explain why UDP-Gal, which has a C4 inverted stereochemistry compared with glucose, shows the lowest affinity for SseK3.

The UDP and GlcNAc present in the SseK3 catalytic pocket are in an arrangement similar to UDP-Glc bound to toxA and the hydrolyzed UDP and Glc in toxB (Fig. S2) (19, 20). Differently from the clostridial toxins, the active site in SseK3 contains a glutamic acid residue, Glu-258, 3.2 Å away from the anomeric carbon. This residue is conserved in all SseK effectors and NleB but is replaced by an isoleucine in toxA, toxB, and LT (19, 20, 31). Previous data suggested an important role of this residue for NleB1 function (22).

### Analysis of UDP-GlcNAc hydrolysis

**Figure 4.**
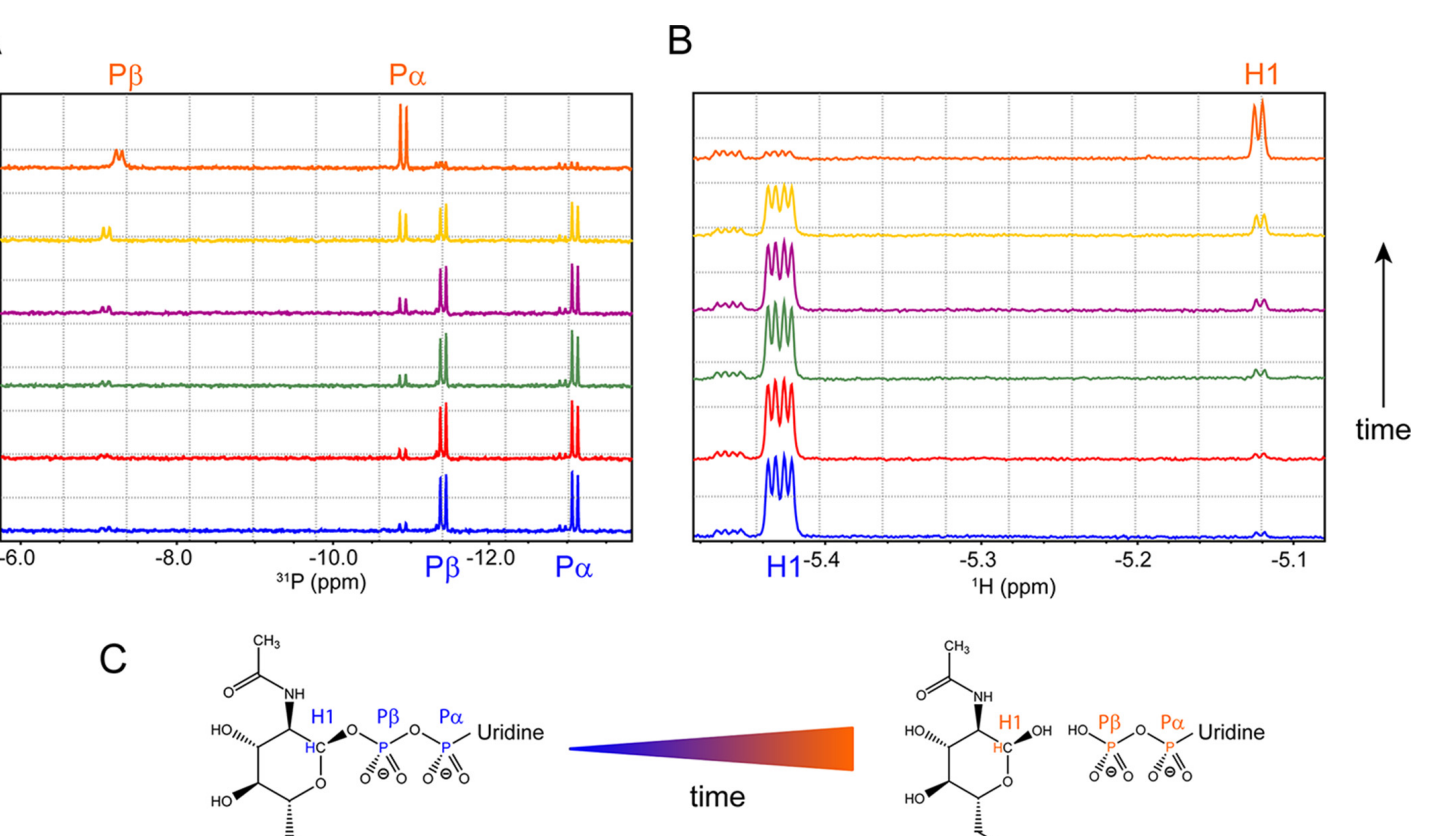
**SseK3(14–335) hydrolyzes UDP-GlcNAc in UDP and free *N*-acetyl α-d-glucosamine.** *A* and *B*, phosphorus (*A*) and proton (*B*) NMR spectra of a 500 μm solution of UDP-GlcNAc in the presence of 10 μm SseK3(14–335). The spectra were recorded at different time points: 0 min (*blue*), 5 min (*red*), 15 min (*green*), 30 min (*purple*), 90 min (*yellow*), and 12 h (*orange*). *C*, UDP-GlcNAc hydrolysis reaction. The Pα and Pβ doublets and the H1 multiplets are assigned in the relative spectra and *highlighted* on the chemical structures of the ligand in its intact and hydrolyzed form.

The presence of hydrolyzed UDP-GlcNAc in the active site shows that, in the absence of a protein substrate, SseK3 can function as a hydrolase. To further explore the ability of SseK3 to hydrolyze UDP-GlcNAc, we used ^31^P and ^1^H NMR spectroscopy. At time 0 (Fig. 4), the 1D ^31^P spectrum of UDP-GlcNAc shows two doublet signals for the Pα, at −13.06 ppm, and Pβ, at −11.41 ppm, for the diphosphate group of the UDP. A well-resolved quadruplet signal system in the 1D proton spectrum of the intact UDP-GlcNAc at 5.43 ppm can also be observed for the anomeric H1 proton of the glucose moiety, resulting from the H1-H2 (3.56 Hz) and H1-Pβ (7.02 Hz) coupling (34, 35). The addition of SseK3(14–335) results in a progressive reduction of both NMR signals with time. The ^31^P spectrum (Fig. 4*A*) shows the emergence of the Pα and Pβ doublet signals from UDP in a free form, whereas the quadruplet signal of the sugar C1 proton (Fig. 4*B*) gradually disappears, substituted by a rising doublet at 5.12 ppm corresponding to the same proton no longer attached to the β-phosphorus of the UDP. The doublet has a ^3^J_H1,H2_ = 3.58 Hz, a value typical for an α-anomeric equatorial–axial stereochemistry for the sugar C1 (the β-form has a value around 7–8 Hz) (36, 37). The chemical shift and proton–proton coupling constant of the glucosamine derived from the hydrolysis of the UDP-GlcNAc are identical to those in the spectrum of free *N*-acetyl α-d-glucosamine. Moreover, mass spectrometry analysis of the reaction mixture does not show SseK3 modification (data not shown), excluding the possibility of intramolecular *N*-GlcNAcylation.

The UDP-GlcNAc hydrolysis proceeds with a retention of the C1 chirality, suggesting that SseK3 is a retaining glycosyltransferase enzyme. Although able to hydrolyze the ligand in the absence of its target substrate, SseK3 does it slowly. There is a reduction of 36 ± 2% of the signal intensities in the ^31^P spectrum for the α- and β-phosphorus nuclei of the diphosphate group of the UDP-GlcNAc after 90 min at 30 °C. The peaks are still visible in the spectrum, albeit very close to the noise level, after the reaction is allowed to proceed for 12 h. UDP-GlcNAc hydrolysis is not observed with SseK3(14–333) (Fig. S3*A*) or in the absence of enzyme (Fig. S3*B*), indicating that together, the C-terminal residues Trp-334 and Arg-335 are necessary for SseK3 functional activity.

### Structure-based analysis of SseK3 function

To test the role of active site residues in the enzymatic activity of SseK3 in a functional framework, multiple amino acid substitutions were tested for their ability to modify the SseK3 substrate TRADD as well as prevent NF-κB activation. We focused on amino acids from our structural analysis of SseK3 (Fig. 3) that are required for coordination of UDP and GlcNAc and hence enzymatic activity. In addition, we were particularly interested to test whether Glu-258 was critical for enzymatic activity, as this residue is only conserved in NleB and SseK glycosyltransferases and has been shown to be required for the function of NleB1 (22).

**Figure 5.**
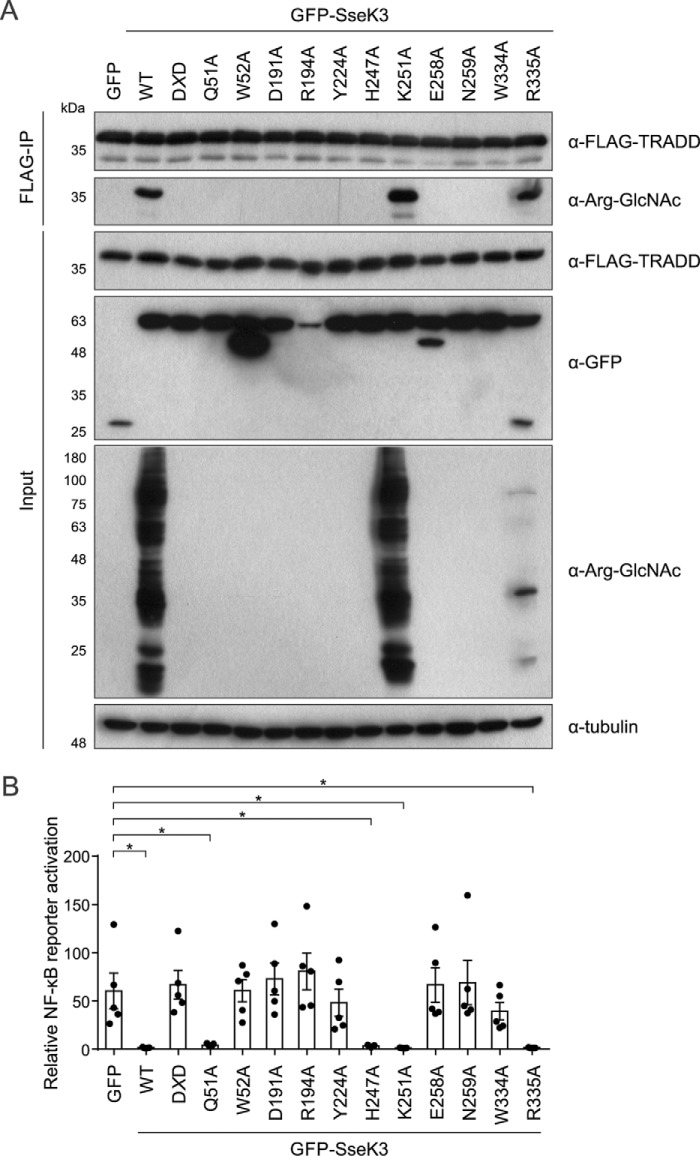
**Screening SseK3 putative catalytic mutants for loss of arginine-GlcNAcylation toward TRADD.** *A*, 293ET cells co-transfected with FLAG-TRADD and the indicated GFP-tagged variants of SseK3 were lysed, and inputs and anti-FLAG immunoprecipitates were analyzed by immunoblotting. Arginine-GlcNAcylation of post-nuclear supernatants (input) and immunoprecipitated FLAG-TRADD was tested using anti-Arg-GlcNAc antibody. Expression of GFP-tagged SseK3 variants was tested using anti-GFP antibody in cell lysates. Antibodies to tubulin were used as a loading control. The shown immunoblots are representative of four independent experiments. *B*, 293ET cells were co-transfected with an NF-κB–dependent luciferase reporter plasmid, pTK-*Renilla* luciferase, and the indicated GFP-tagged SseK3 mutants for 24 h before overnight stimulation with 50 ng/ml TNFα. Luciferase activity was measured in cell lysates, and results are presented as fold activation relative to unstimulated controls expressing each SseK3 variant. Data are the mean ± S.E. of five independent experiments, for which individual data points are indicated. Cell lysates from *B* were analyzed by immunoblotting in Fig. S4. *, *p* < 0.05 one-way analysis of variance. D*X*D corresponds to the SseK3 D226A/D228A mutant.

Individual SseK3 mutants were co-transfected into 293ET cells together with FLAG-TRADD, and following anti-FLAG immunoprecipitation, the GlcNAcylation of TRADD was analyzed by immunoblotting. As a control, transfected SseK3 K251A remained active like wildtype SseK3. In contrast, and as expected, mutation of the manganese-coordinating D*X*D (D226A/D228A) motif ablated SseK3-induced arginine GlcNAcylation in cell lysates and the modification of immunoprecipitated TRADD (Fig. 5*A*) (8). The conserved glutamate (Glu-258, predicted to coordinate GlcNAc), which is present in SseK and NleB proteins but not other structurally related glycosyltransferases, was also essential for arginine GlcNAcylation of TRADD. In addition, mutations predicted to be required for coordination of uracil (W52A and Y224A) and GlcNAc (D191A, N259A, and W334A) also eliminated SseK3-induced protein arginine GlcNAcylation, and, more specifically, no modification of TRADD was detected (Fig. 5*A*). Transfected SseK3 R194A (predicted to disrupt GlcNAc binding) appeared inactive, but this mutant was not stably expressed and should be approached with caution (Fig. 5*A*). Surprisingly, SseK3 with a mutation in the C-terminal residue Arg-335 (to alanine) retained the ability to modify numerous proteins, including TRADD. However, the pattern of GlcNAcylation was different, with significantly fewer proteins modified when compared with wildtype SseK3 (Fig. 5*A*).

Next, to test whether enzymatic activity correlated with the ability of SseK3 to inhibit TNFα-induced NF-κB activation, variants of SseK3 were transfected into 293ET cells together with an NF-κB–dependent luciferase reporter plasmid. We predicted that Q51A would still be catalytically active, as it is the backbone carbonyl group and not the side chain that is involved in interactions with the ligand (Fig. 2*C*). Interestingly, whereas we were not able to detect arginine GlcNAcylation of TRADD, SseK3 Q51A was still a potent inhibitor of NF-κB activation (Fig. 5), suggesting that catalytic activity is retained but greatly reduced below the level of detection. The side-chain Nϵ of residue His-247 is 4.4 Å away from the oxygen of the GlcNAc acetyl group, yet a similar finding was observed for SseK3 H247A, with inhibition of NF-κB activation occurring without detectable modification of TRADD (Fig. 5). This suggests that very low and non-detectable levels of arginine GlcNAcylation are sufficient for SseK3-mediated inhibition of NF-κB activation, presumably due to the irreversible nature of the arginine modification. As the rest of the mutations in SseK3 that ablated the ability of the enzyme to arginine-GlcNAcylate TRADD (D*X*D, W52A, Y224A, D191A, E258A, N259A, and W334A) also caused an inability of SseK3 to inhibit TNFα-induced NF-κB activation (Fig. 5*B* and Fig. S4), these data reveal that enzymatic activity is required for SseK3 function.

## Discussion

**Figure 6.**
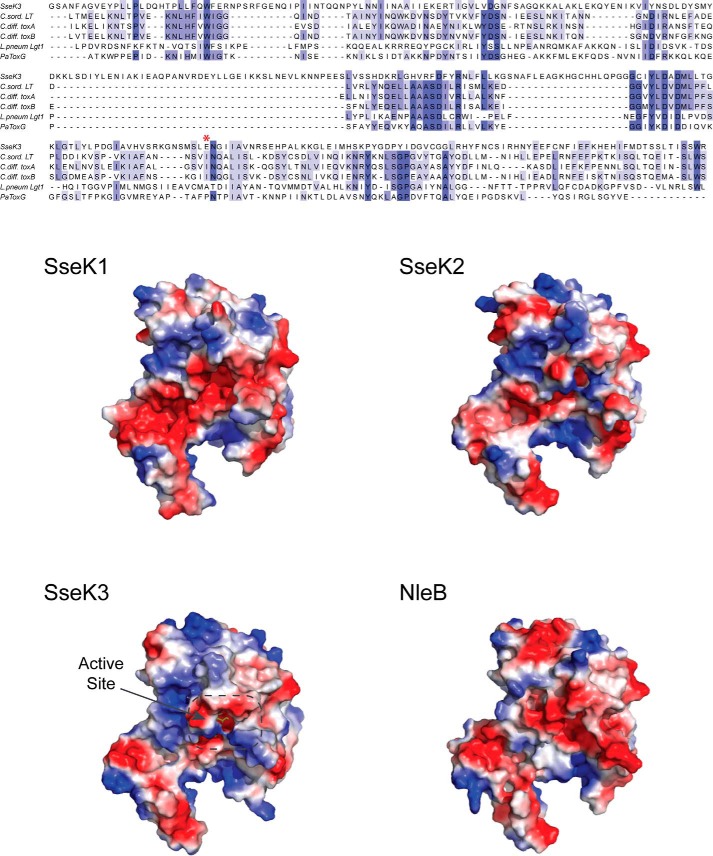
**Glycosyltransferase structural alignment and SseK1, SseK2, and NleB1 homology structural models.** *Top*, structural alignment of SseK3 with *C. sordelli* LT (PDB code 2VKD, 19% sequence identity), *C. difficile* toxin A (3SRZ, 17%), toxin B (2BVL, 15%), *L. pneumophila* Lgt1 (3JSZ, 11%), and *P. asymbiotica* toxin (4MIX, 19%). The catalytically important Glu-258 residue is marked with an *asterisk* in the alignment. *Bottom*, solvent-accessible surface representation *colored* according to the electrostatic potential (*blue*, positive; *red*, negative) of the structure of SseK3(14–335) bound to its UDP-GlcNAc ligand and homology structural models of SseK1 (55% sequence identity with SseK3), Ssek2 (72%), and NleB1 (53%). Ligand bound in the active site is *highlighted*.

Here we report the structure of the *Salmonella* arginine *N*-glycosyltransferase, SseK3, in its apo and ligand-bound forms. The protein belongs to the GT-A family of glycosyltransferase enzymes and binds the ligand in a metal ion–dependent manner via a D*X*D motif. The dynamic features of the last 15 residues of SseK3 are important, as they experience a large conformational change upon ligand and metal binding. As suggested by solution NMR spectroscopy, the C-terminal residues are disordered in the unbound SseK3 structure, with structural reorganization following primary substrate binding, with an open-close catalytic cycle that, as in the case of a number of other glycosyltransferases, is likely to support SseK3 enzymatic activity (38, 39). Residue Trp-334 in the active site pocket occupies the same structural position as Trp-520 in the catalytic fragment of the clostridial lethal toxin in complex with UDP-Glc and manganese (PDB code 2VKD) (31), and it is conserved in toxin A (Trp-519) (20) and toxin B (Trp-520) (19) of the same organism (Fig. 6 and Fig. S2). A tryptophan residue (Trp-520) is also present in the active site of the *L. pneumophila* Lgt1 in complex with UDP-Glc and magnesium (PDB code 3JSZ) (21) and in a structurally equivalent position in the unbound form of the α-toxin from *Clostridium novyi* (PDB code 2VK9) (31). As for the other toxins, Trp-334 is a pivotal residue in the opening and closure of the catalytic cleft (40). In the ligand-bound SseK3, its Nϵ1 forms a hydrogen bond with the UDP Pβ oxygen, whereas the equivalent tryptophan residue (Trp-519) in the structure of toxin A hydrogen-bonds the glycosidic oxygen of the intact UDP-Glc (Fig. S2*B*) (31). Trp-334 is therefore in a position to stabilize the emerging negative charge of the former glycosidic oxygen transferred onto the UDP. Once the enzymatic reaction has occurred, the opening of the catalytic site and the release of the UDP reinitiate the cascade; indeed, mutation of Trp-334 ablates the catalytic and functional activity of SseK3.

The presence of a hydrolyzed UDP-GlcNAc in the protein active site suggests that SseK3 can function as a hydrolase, a process observed also in the case of toxin A (28) and toxin B (19). The hydrolysis of UDP-GlcNAc, in the absence of target substrate, is likely to be a proxy of its physiological glycosyltransferase activity, and the release observed by NMR of an α-anomeric form of GlcNAc, as well as the presence of the same molecule in the enzyme active site, indicates that SseK3 is likely to be a retaining *N*-glycosylating enzyme.

Reaction mechanisms of retaining glycosyltransferases have been controversial, but it is generally accepted that they operate through the stabilization of an oxocarbenium-like transition state (41). Depending on the presence of a nucleophile in the active site correctly positioned on the β-face of the donor substrate, the reaction could proceed either via a front-face or double-inversion mechanism (42). For the clostridial *O*-glycosylating toxins, in the absence of other available bases in the active site, the deprotonation of the acceptor nucleophile is carried out by the nucleotide Pβ oxygen. Following the formation of a transient oxocarbenium state, the departure of the leaving sugar O1 and the formation of the new glycosidic bond occur on the same side of the anomeric carbon, retaining its stereochemistry (31). In the double-displacement reaction, the presence of a nucleophile in close proximity to the anomeric carbon, directly under the sugar substrate β-face, stabilizes an oxocarbenium-like enzyme–ligand adduct. The reaction is then followed by a subsequent direct substitution by the target nucleophile at the opposite site of the anomeric C1 with a double-inversion mechanism that retains the original carbon stereochemistry. This is the case for lysozyme (43) and GT6 family member α3GalT (44). For these enzymes, a well-located nucleophile within the active site (Asp-52 for lysozyme and Glu-317 for α3GalT) is a necessary element for the creation of a stereochemically inverted intermediate set for nucleophilic substitution. Despite their structural similarity, the presence of a glutamate side chain in the active site of SseK3, 3.2 Å from the anomeric carbon, suggests that this enzyme could operate as a retaining GT more similarly to α3GalT than to the clostridial toxins. The Glu-258 side chain is important for SseK3 catalytic activity and is a conserved active-site residue across the SseK effectors family and in NleB1, substituted by uncharged residues in structurally related toxins (Fig. 6). Mutation into an alanine residue, similarly to the mutation of the equivalent residue in NleB1 (E253A), impairs the arginine GlcNAcylation ability of SseK3 and NleB1 without, for the latter, abrogating binding to the target protein (22). To date, the only available structure of an arginine-glycosylation enzyme is that of the *Pseudomonas aeruginosa* EarP that catalyzes the transfer of a rhamnose molecule onto the bacterial translation elongation factor EF-P (16). EarP is an inverting glycosyltransferase belonging to the GT-B family and structurally unrelated to SseK3. Interestingly, EarP has three negatively charged residues in the active site (Asp-13, Asp-17, and Glu-273), in close proximity to the sugar, that have been shown to be important in the catalytic activity and play a crucial role in the stabilization of the positive charge of the acceptor guanidino group. Mutation of those residues to alanine, as for the E258A mutation in SseK3, severely impairs EarP enzymatic activity without disrupting substrate binding (16).

We therefore propose a catalytic mechanism, in which the glutamate side chain of Glu-258 acts as the intramolecular nucleophile in the first step of a double-displacement mechanism, forming an SseK3-GlcNAc intermediate state primed for the C1 nucleophilic attack to the sugar α-side by the guanidino group on the target arginine. Glu-258 could aid in the selection of the arginine as the *N*-GlcNAcylation site and, similarly to the phosphate role in the clostridial toxins, increase the nucleophilic character of the acceptor by deprotonation of its side chain. A double-displacement mechanism, where the C1-O1 bond is broken before the nucleophilic attack, could also be favored by the presence of the guanidino group of Arg-335 tucked into the SseK3 active site. Arg-335 sits above the sugar O1, obstructing the access to the sugar α-side, sterically hindering a direct front-face reaction.

In SseK3, the well-located Arg-335 side chain hints at a potential intramolecular GlcNAcylation process. However, *in vitro* auto-GlcNAcylation of SseK3(14–335) is unlikely, as NMR and mass spectrometry experiments only detected unbound glucosamine following UDP-GlcNAc hydrolysis by SseK3. Nevertheless, a specific role of this residue in SseK3 function cannot be excluded. In the *E. coli* MurG, an *O*-GlcNAc GT-B family glycosyltransferase, the presence of a positively charged arginine residue (Arg-261) in the active pocket provides extra stability for the negatively charged UDP emerging from the catalytic reaction (45). In the structure of the UDP and GlcNAc-bound GtfA, an *O*-GlcNAc GT-B, an active-site arginine residue (Arg-328), crucial for catalytic activity, forms a strong hydrogen bond with the Pβ oxygen, stabilizing the negative charge generated from the processing of the substrate (33). The active site of EarP has an arginine residue (Arg-271), whose guanidino Nϵ1 is 2.5 Å from the phosphate oxygen that participates in the stabilization of the enzymatic products whose mutation into an alanine reduces EarP glycosylation activity (16). In SseK3, Arg-335 is tucked in the active site at 2.62 Å from the sugar O1 and 3.24 Å from the oxygen β-phosphate, and its mutation R335A induced a severely reduced arginine-GlcNAcylation pattern compared with wildtype SseK3 (Fig. 5). As for EarP, this could reflect a decrease in enzymatic activity due to a decreased stabilization of the enzymatic products. Alternatively, as this residue is only conserved in SseK2, which like SseK3 localizes to the Golgi network during infection (8), but is substituted by an alanine in SseK1 and absent in NleB1, it could be indirectly involved in substrate specificity via reduced Golgi network association.

During infection of macrophages, similarly to their *E. coli* ortholog, SseK1 and SseK3 both function to mediate inhibition of NF-κB activation as well as inhibition of an inflammatory necroptotic host cell death (8). The structure of SseK3 bound to the hydrolysis products of its donor substrate revealed which amino acids are necessary for ligand binding and hence arginine GlcNAcylation of TRADD and SseK3-mediated inhibition of NF-κB activity. In addition to the ion-coordinating D*X*D motif, previous mutational analysis of NleB1 identified residue Tyr-219 as required for function (22). This amino acid is conserved in SseK proteins (Tyr-224) and is important for the coordination of the uracil of the UDP and therefore necessary for both SseK3 catalytic activity and function.

SseK effector proteins share a high sequence identity, but most of the conserved residues are those in the catalytic domain (Fig. 1 and Fig. S5). Sequence variability is mainly concentrated around residues in the α-helical insertion, suggesting that this region might direct substrate specificity. Despite its similarity to the other effectors, SseK2 does not show activity in cell-based assays (11). However, purified SseK2 can arginine-GlcNAcylate FADD *in vitro*, without forming a stable interaction, suggesting that this enzyme is functional (11). As all residues tested that are required for enzymatic activity in SseK3 are conserved in SseK2, it is not immediately clear why SseK2 is not active after translocation into host cells. In the absence of structural information for the other members of the SseK family, we created homology models of SseK1, SseK2, and NleB1 based on the SseK3 crystal structure (Fig. 6). The distribution of surface charges shows that both SseK1 and NleB1 have the active site surrounded by acidic residues, whereas the surfaces of SseK2 and SseK3 appear to be more similar, with 72% sequence identity, having the ligand site surrounded by larger positively charged areas compared with SseK1. Also, as the C-terminal residue in both SseK2 and SseK3 is a positively charged residue (Arg-348 and Arg-335), substituted by a smaller side chain in SseK1 (Ala-332) and absent in NleB1, the active site in SseK1 and NleB1 appears to be more accessible than in SseK2 and SseK3. Recently, it has been reported that NleB1 preferentially GlcNAcylates the death domain (DD) of FADD at residue Arg-117 (23), although it can interact with and modify the DD of other proteins, including TRADD and RIPK1 (9, 10). Interestingly, SseK1 is the only SseK family member that can modify FADD (also at Arg-117) after bacterial delivery into host cells (8). The structure of FADD-DD shows an extensive basic surface patch whose residues have been shown to be important in the interaction with Fas receptor (CD95) (46). In particular, mutation of Arg-117, part of the FADD-DD extended positively charged surface, has been shown to abolish interaction with the cytoplasmic death domain of CD95 (47). Therefore, the contiguous acidic surfaces of SseK1 and NleB1 could act as a complementary surface, binding the death domain of FADD and allowing the side chain of residue Arg-117 to position itself in the active site for sugar transfer. The lack of activity in cell-based assays of SseK3 toward FADD and the absence of a phenotype in *Salmonella* for SseK2 could then be the result of the interplay between a less accessible active site and altered substrate specificity, with SseK3 still able to modify TRADD.

In summary, we have solved the first X-ray crystal structure of a GT-A arginine glycosyltransferase, identifying core-conserved residues that are required for catalytic activity and virulence function of SseK3, allowing us to propose a potential enzymatic mechanism for *N*-arginine glycosylation.

## Experimental procedures

### Protein cloning, expression, and purification

Wildtype SseK3(14–333) and SseK3(14–335) constructs (Uniprot: A0A0H3NMP8) were cloned in pGEX-6P1 (GE Healthcare) vector by the standard Gibson assembly protocol (48) and expressed with a cleavable N-terminal GST tag. The proteins were expressed in *E. coli* BL21 (DE3) Gold in LB supplemented with 100 μg/ml ampicillin. Cells were grown at 30 °C until *A*_600_ ≈ 0.6–0.8, induced with 0.5 mm isopropyl β-d-1-thiogalactopyranoside, and incubated at 18 °C for overnight expression. Proteins were purified by GST-Sepharose affinity chromatography followed by cleavage of the GST N-terminal tag by 3C-protease and size-exclusion chromatography in 25 mm HEPES buffer, pH 7.5, 150 mm NaCl, and 0.5 mm TCEP. The 3C-cleavage leaves amino acids GPLGS preceding the first amino acid of SseK3. SseK3(14–335) purification was performed at 4 °C, in the presence of EDTA-free protease inhibitor mixture and 1 mm phenylmethylsulfonyl fluoride in the lysis buffer. Selenomethionine labeling of SseK3(14–333) was achieved by using the Molecular Dimensions standard labeling protocol of M9 supplemented with l-selenomethionine with the auxotrophic strain *E. coli* 834 (DE3) (49). SseK3(14–333) and SseK3(14–335) concentrations were calculated using the values for ϵ at λ = 280 nm of 29,340 and 34,840 m^−1^ cm^−1^, respectively. Mass spectrometry was used to check selenomethionine incorporation and protein molecular masses. The server SWISS-MODEL was used to create structural homology models of SseK1, SseK2, and NleB1 (50).

### SseK3 mutant plasmid construction

SseK3 mutant variants were created by overlap mutagenesis PCR from the m4pGFP-SseK3 wildtype plasmid (8) and ligated into the mammalian expression vector m4pGFP to create plasmids encoding SseK3 mutants with an N-terminal GFP tag. All plasmids were checked by sequencing.

### Cell culture

293ET cells (gift from Felix Randow) were cultured in Dulbecco's modified Eagle's medium (Sigma) supplemented with 10% fetal calf serum (Sigma) at 37 °C in 5% CO_2_.

### Isothermal titration calorimetry

ITC experiments were performed at 293 K using a Microcal iTC200 calorimeter (Malvern). The protein solutions were prepared in buffer containing 25 mm HEPES buffer, pH 7.5, 150 mm NaCl, 0.5 mm TCEP, and 5 mm either MgCl_2_ or MnCl_2_. All experiments were performed by placing the solution containing SseK3 in the cell at concentrations of 50 μm and the solution containing the UDP-glucose derivatives in the syringe at 500 μm. The concentrations of UDP derivatives were estimated using the UDP extinction coefficient at λ = 262 nm of 9800 m^−1^ cm^−1^. For each titration 20 injections of 2 μl were performed. Integrated data, corrected for heats of dilution, were fitted using a nonlinear least-squares algorithm to a 1:1 binding model, using the MicroCal Origin version 7.0 software package. The fitting parameters are Δ*H* (reaction enthalpy change in kcal mol^−1^), *K_b_* (equilibrium binding constant in m^−1^), and *n* (number of binding sites). The entropic contribution values (defined as −*T*Δ*S*) were calculated from the values of Δ*H* and *K_b_*. Each experiment was repeated at least twice, and average values are reported in Table 1.

### Crystallization

Initial screens for SseK3(14–333) were set up by the sitting-drop method at two protein concentrations (20 and 10 mg/ml) by combining 0.1 μl of protein solution with 0.1 μl of reservoir. Commercially available crystallization screens were dispensed by using an automated Mosquito machine (TTP Labtech). The most promising initial crystal hits grew from drops set up with 10 mg/ml protein solution in 0.1 m Tris, pH 8.5, 0.2 m NaCl, and 25% PEG 3350. No single crystals were obtained from the initial screening, and multiple rounds of seeding were necessary to improve crystal quality. The seed stock was prepared by harvesting the small crystals, obtained in 0.1 m Tris, pH 8.5, 0.2 m NaCl, and 25% PEG 3350, in 50 μl of solution of the same condition. The stock was then homogenized by vortexing for 30 s using a Hampton seed bead. Sitting drops were dispensed by mixing 0.2 μl of protein solution at 9 mg/ml, 0.18 μl of reservoir solution (0.1 m Tris pH 8.5, 0.2 m NaCl, and 25% PEG 3350), and 0.02 μl of seed stock. Single crystals of better diffraction quality could be grown with the selenomethionine SseK3(14–333) protein sample in the same condition but using a total protein concentration of 7 mg/ml. For X-ray data acquisition, crystals were cryoprotected with mother liquor containing 0.1 m Tris, pH 8.5, 0.2 m NaCl, 25% PEG 3350, and 20% ethylene glycol.

To crystallize SseK3(14–335) in complex with UDP-GlcNAc, a stock solution was prepared by mixing the protein at 10 mg/ml with a 3-fold molar excess of UDP-GlcNac (900 μm) and a final concentration of MnCl_2_ of 5 mm. Small single crystals were obtained in 0.1 m Tris, pH 8.0, 0.2 m NaI, and 20% PEG 3350 and improved by multiple rounds of seeding. For X-ray data acquisition, crystals were cryoprotected with mother liquor containing 0.1 m Tris, pH 8.0, 0.2 m NaI, 20% PEG 3350, and 30% trehalose. All of the crystallization experiments were done at 20 °C.

### Data collection, phasing, and refinement

Despite keeping crystallization and cryoprotection protocols the same, in total, four polymorphs were observed for SeMet-labeled SseK3(14–333) crystals. Data from a monoclinic crystal were collected at the long-wavelength MX beamline I23 at Diamond Light Source (29) for experimental SAD phasing using a wavelength of λ = 2.7751 Å and processed with XDS (51). The anomalous substructure of 20 selenium and 20 sulfur atoms (corresponding to four molecules in the asymmetric unit) was located by SHELXD (52) at 3.5 Å resolution. The substructure solution was confirmed by the presence of a 4-fold non-crystallographic symmetry. Initial experimental electron density maps were of poor quality, possibly due to the presence of a pseudotranslation and the anisotropy of the data. Because automatic building programs failed to provide a starting model, a careful inspection of the density map revealed the position of two helices that were placed as polyalanine helices using Coot (53). The 4-fold non-crystallographic symmetry was applied to locate the corresponding helices in the asymmetric unit. The anomalous substructure and the initial model of the asymmetric unit comprising eight helices were fed to CRANK2 (54) to produce improved maps benefiting from 4-fold averaging and an almost complete model.

Complete high-resolution data from for SeMet-labeled crystals of SseK3(14–333) were collected at the Swiss Light Source (Villigen, Switzerland) and processed using DIALS (http://dials.diamond.ac.uk/).^5^ The structure of SeMet-labeled SseK3(14–333) was solved at a single wavelength of 0.97640 Å using the structural model obtained by long-wavelength S-SAD as template in a molecular replacement with MOLREP (55). Data for SseK3(14–335) bound to UDP-GlcNAc and Mn^2+^ were collected at Diamond Light Source, I04 (Oxford, UK) and processed using XDS (51). The structure of SseK3(14–335) co-crystallized in the presence of UDP-GlcNAc and Mn^2+^ was solved using the apo-structure as template for molecular replacement with MOLREP (55).

Models were iteratively improved by manual building in Coot (53) and refined using REFMAC5 (56) and Phenix (57). The parameters for the ligand stereochemistry were obtained from the standard Coot library. All structural figures were prepared in PyMOL (Schrödinger, LLC) and Chimera (58). Further details on data collection and refinement statistics are summarized in Table 2.

### NF-κB reporter assays

1 × 10^5^ 293ET cells (seeded the day before use) were transfected with a mixture of 50 ng of p4kB:Luc, 20 ng of pRLTK, and 500 ng of m4pGFP-SseK3 variants (or m6pPAC-FLAG-GFP control) using Lipofectamine 2000 (Invitrogen). After 24 h of transfection, the cells were stimulated with 50 ng/ml human TNFα (Sigma) for 17 h, and luciferase activity was measured using the Dual-Luciferase reporter assay system (Promega) and a Tecan Infinite200 PRO plate reader. NF-κB-regulated luciferase activity was first normalized to *Renilla* luciferase activity, and then the fold activation relative to unstimulated conditions of each SseK3 variant was calculated.

### TRADD modification by SseK3 variants

4 × 10^5^ 293ET cells were transfected for 40 h with 1 μg of m6pPAC-FLAG-TRADD and 1 μg of m4pGFP-SseK3 variant (or 1 μg of m6pPAC-FLAG-GFP control) plasmid DNA using Lipofectamine 2000 (Invitrogen). Subsequently, the cells were harvested and lysed for 30 min on ice in lysis buffer (150 mm NaCl, 0.3% (v/v) Triton X-100, 20 mm Tris-Cl (pH 7.4), 5% glycerol, 5 mm EDTA, 1 mm phenylmethylsulfonyl fluoride, 1 mm benzamide, 2 μg/ml aprotinin, 5 μg/ml leupeptin, 1 mm DTT), and cytoplasmic proteins were isolated by a 30-min centrifugation at 16,000 × *g* at 4 °C. FLAG-TRADD was then immunoprecipitated using anti-FLAG M2 Affinity Gel (Sigma) for 2 h at 4 °C, washed, and analyzed by SDS-PAGE and immunoblotting using rabbit anti-FLAG (Sigma), rabbit anti-GFP (Invitrogen), rabbit anti-arginine-GlcNAc (EPR18251, Abcam), and rabbit anti-tubulin (EPR16774, Abcam) antibodies and horseradish peroxidase–conjugated anti-rabbit secondary antibody (Dako) for detection.

### Nuclear magnetic resonance

All spectra were recorded at 25 °C on a Bruker AVANCE spectrometer operating at 700 MHz, equipped with a cryogenically cooled quadruple-resonance (^1^H, ^15^N, ^13^C, and ^31^P) probe including *z*-axis pulse field gradients. Data were acquired and processed with Topspin version 3.5 (Bruker). Reaction solutions of 10 μm of both SseK3 constructs (Santa Cruz Biotechnology, Inc.; weight >98%) were prepared in 20 mm Tris, pH 7.5, 100 mm NaCl, 0.5 mm TCEP, and 5 mm MgCl_2_. 500 μm UDP-GlcNAc was added to the protein solutions, and hydrolysis was allowed to proceed at 30 °C under gentle stirring. The reaction was quenched at different time points by adding 10 mm EDTA (pH 7.5) and 5% D_2_O required for the magnetic field lock. The same reaction conditions were used to monitor UDP-GlcNAc hydrolysis in the absence of enzyme. 1D ^1^H and ^31^P NMR spectra were recorded using the standard excitation sculpting pulse sequences zgesp and zgpg30 implemented in Topspin acquisition software.

## Author contributions

D. E., T. L. T., and K. R. conceptualization; D. E., R. A. G., L. M., K. E. O., and A. W. formal analysis; D. E., R. A. G., L. M., K. E. O., and A. W. investigation; D. E. and T. L. T. writing-original draft; D. E., R. A. G., L. M., A. W., T. L. T., and K. R. writing-review and editing; T. L. T. and K. R. supervision; T. L. T. and K. R. funding acquisition.

## Supplementary Material

Supporting Information

## Footnotes

5 Please note that the JBC is not responsible for the long-term archiving and maintenance of this site or any other third party hosted site.

## Footnotes

4 The abbreviations used are:

SPI-2: *Salmonella* pathogenicity island 2
T3SS: type III secretion system
GlcNAc: *N*-acetylglucosamine
TRADD: TNFR1-associated death domain protein
FADD: FAS-associated protein with death domain
EF-P: elongation factor P
ITC: isothermal titration calorimetry
a.u.: asymmetric unit
RMSD: root mean square deviation
LT: lethal toxin
toxA and toxB: toxin A and toxin B, respectively
DD: death domain
TCEP: tris(2-carboxyethyl)phosphine
SAD: single-wavelength anomalous dispersion
SeMet: selenomethionine.

## References

[1] Coburn B., Grassl G. A., and Finlay B. B. (2007) *Salmonella*, the host and disease: a brief review. Immunol. Cell Biol. 85, 112–118 10.1038/sj.icb.7100007 17146467

[2] Feasey N. A., Dougan G., Kingsley R. A., Heyderman R. S., and Gordon M. A. (2012) Invasive non-typhoidal salmonella disease: an emerging and neglected tropical disease in Africa. Lancet 379, 2489–2499 10.1016/S0140-6736(11)61752-2 22587967 PMC3402672

[3] Jennings E., Thurston T. L. M., and Holden D. W. (2017) *Salmonella* SPI-2 type III secretion system effectors: molecular mechanisms and physiological consequences. Cell Host Microbe 22, 217–231 10.1016/j.chom.2017.07.009 28799907

[4] Ochman H., Soncini F. C., Solomon F., and Groisman E. A. (1996) Identification of a pathogenicity island required for *Salmonella* survival in host cells. Proc. Natl. Acad. Sci. U.S.A. 93, 7800–7804 10.1073/pnas.93.15.7800 8755556 PMC38828

[5] Hensel M., Shea J. E., Gleeson C., Jones M. D., Dalton E., and Holden D. W. (1995) Simultaneous identification of bacterial virulence genes by negative selection. Science 269, 400–403 10.1126/science.7618105 7618105

[6] Rahman M. M., and McFadden G. (2011) Modulation of NF-κB signalling by microbial pathogens. Nat. Rev. Microbiol 9, 291–306 10.1038/nrmicro2539 21383764 PMC3611960

[7] Brown N. F., Coombes B. K., Bishop J. L., Wickham M. E., Lowden M. J., Gal-Mor O., Goode D. L., Boyle E. C., Sanderson K. L., and Finlay B. B. (2011) Salmonella phage ST64B encodes a member of the SseK/NleB effector family. PLoS One 6, e17824 10.1371/journal.pone.0017824 21445262 PMC3060822

[8] Günster R. A., Matthews S. A., Holden D. W., and Thurston T. L. (2017) SseK1 and SseK3 type III secretion system effectors inhibit NF-κB signaling and necroptotic cell death in *Salmonella*-infected macrophages. Infect. Immun. 85, e00010–17 28069818 10.1128/IAI.00010-17 PMC5328493

[9] Pearson J. S., Giogha C., Ong S. Y., Kennedy C. L., Kelly M., Robinson K. S., Lung T. W., Mansell A., Riedmaier P., Oates C. V., Zaid A., Mühlen S., Crepin V. F., Marches O., Ang C. S., et al (2013) A type III effector antagonizes death receptor signalling during bacterial gut infection. Nature 501, 247–251 10.1038/nature12524 24025841 PMC3836246

[10] Li S., Zhang L., Yao Q., Li L., Dong N., Rong J., Gao W., Ding X., Sun L., Chen X., Chen S., and Shao F. (2013) Pathogen blocks host death receptor signalling by arginine GlcNAcylation of death domains. Nature 501, 242–246 10.1038/nature12436 23955153

[11] El Qaidi S., Chen K., Halim A., Siukstaite L., Rueter C., Hurtado-Guerrero R., Clausen H., and Hardwidge P. R. (2017) NleB/SseK effectors from *Citrobacter rodentium*, *Escherichia coli*, and *Salmonella enterica* display distinct differences in host substrate specificity. J. Biol. Chem. 292, 11423–11430 10.1074/jbc.M117.790675 28522607 PMC5500807

[12] Hart G. W., Slawson C., Ramirez-Correa G., and Lagerlof O. (2011) Cross talk between *O*-GlcNAcylation and phosphorylation: roles in signaling, transcription, and chronic disease. Annu. Rev. Biochem. 80, 825–858 10.1146/annurev-biochem-060608-102511 21391816 PMC3294376

[13] Pan M., Li S., Li X., Shao F., Liu L., and Hu H. G. (2014) Synthesis of and specific antibody generation for glycopeptides with arginine *N*-GlcNAcylation. Angew. Chem. Int. Ed. Engl. 53, 14517–14521 10.1002/anie.201407824 25353391

[14] Lairson L. L., Henrissat B., Davies G. J., and Withers S. G. (2008) Glycosyltransferases: structures, functions, and mechanisms. Annu. Rev. Biochem. 77, 521–555 10.1146/annurev.biochem.76.061005.092322 18518825

[15] Lassak J., Keilhauer E. C., Fürst M., Wuichet K., Gödeke J., Starosta A. L., Chen J. M., Søgaard-Andersen L., Rohr J., Wilson D. N., Häussler S., Mann M., and Jung K. (2015) Arginine-rhamnosylation as new strategy to activate translation elongation factor P. Nat. Chem. Biol. 11, 266–270 10.1038/nchembio.1751 25686373 PMC4451828

[16] Krafczyk R., Macošek J., Jagtap P. K. A., Gast D., Wunder S., Mitra P., Jha A. K., Rohr J., Hoffmann-Röder A., Jung K., Hennig J., and Lassak J. (2017) Structural basis for EarP-mediated arginine glycosylation of translation elongation factor EF-P. MBio 8, e01412–17 28951478 10.1128/mBio.01412-17 PMC5615199

[17] Singh D. G., Lomako J., Lomako W. M., Whelan W. J., Meyer H. E., Serwe M., and Metzger J. W. (1995) β-Glucosylarginine: a new glucose-protein bond in a self-glucosylating protein from sweet corn. FEBS Lett. 376, 61–64 10.1016/0014-5793(95)01247-6 8521968

[18] Jank T., Bogdanović X., Wirth C., Haaf E., Spoerner M., Böhmer K. E., Steinemann M., Orth J. H., Kalbitzer H. R., Warscheid B., Hunte C., and Aktories K. (2013) A bacterial toxin catalyzing tyrosine glycosylation of Rho and deamidation of G_q_ and G_i_ proteins. Nat. Struct. Mol. Biol. 20, 1273–1280 10.1038/nsmb.2688 24141704

[19] Reinert D. J., Jank T., Aktories K., and Schulz G. E. (2005) Structural basis for the function of *Clostridium difficile* toxin B. J. Mol. Biol. 351, 973–981 10.1016/j.jmb.2005.06.071 16054646

[20] Pruitt R. N., Chumbler N. M., Rutherford S. A., Farrow M. A., Friedman D. B., Spiller B., and Lacy D. B. (2012) Structural determinants of *Clostridium difficile* toxin A glucosyltransferase activity. J. Biol. Chem. 287, 8013–8020 10.1074/jbc.M111.298414 22267739 PMC3318759

[21] Lü W., Du J., Stahl M., Tzivelekidis T., Belyi Y., Gerhardt S., Aktories K., and Einsle O. (2010) Structural basis of the action of glucosyltransferase Lgt1 from *Legionella pneumophila*. J. Mol. Biol. 396, 321–331 10.1016/j.jmb.2009.11.044 19941871

[22] Wong Fok Lung T., Giogha C., Creuzburg K., Ong S. Y., Pollock G. L., Zhang Y., Fung K. Y., Pearson J. S., and Hartland E. L. (2016) Mutagenesis and functional analysis of the bacterial arginine glycosyltransferase effector NleB1 from enteropathogenic *Escherichia coli*. Infect. Immun. 84, 1346–1360 10.1128/IAI.01523-15 26883593 PMC4862703

[23] Scott N. E., Giogha C., Pollock G. L., Kennedy C. L., Webb A. I., Williamson N. A., Pearson J. S., and Hartland E. L. (2017) The bacterial arginine glycosyltransferase effector NleB preferentially modifies Fas-associated death domain protein (FADD). J. Biol. Chem. 292, 17337–17350 10.1074/jbc.M117.805036 28860194 PMC5655511

[24] Karavolos M. H., Roe A. J., Wilson M., Henderson J., Lee J. J., Gally D. L., and Khan C. M. (2005) Type III secretion of the *Salmonella effector* protein SopE is mediated via an N-terminal amino acid signal and not an mRNA sequence. J. Bacteriol. 187, 1559–1567 10.1128/JB.187.5.1559-1567.2005 15716426 PMC1064012

[25] Ghosh P. (2004) Process of protein transport by the type III secretion system. Microbiol. Mol. Biol. Rev. 68, 771–795 10.1128/MMBR.68.4.771-795.2004 15590783 PMC539011

[26] Li W., Liu Y., Sheng X., Yin P., Hu F., Liu Y., Chen C., Li Q., Yan C., and Wang J. (2014) Structure and mechanism of a type III secretion protease, NleC. Acta Crystallogr. D Biol. Crystallogr. 70, 40–47 10.1107/S1399004713024619 24419377

[27] Yao Q., Zhang L., Wan X., Chen J., Hu L., Ding X., Li L., Karar J., Peng H., Chen S., Huang N., Rauscher F. J. 3rd, and Shao F. (2014) Structure and specificity of the bacterial cysteine methyltransferase effector NleE suggests a novel substrate in human DNA repair pathway. PLoS Pathog. 10, e1004522 10.1371/journal.ppat.1004522 25412445 PMC4239114

[28] D'Urzo N., Malito E., Biancucci M., Bottomley M. J., Maione D., Scarselli M., and Martinelli M. (2012) The structure of *Clostridium difficile* toxin A glucosyltransferase domain bound to Mn^2+^ and UDP provides insights into glucosyltransferase activity and product release. FEBS J. 279, 3085–3097 10.1111/j.1742-4658.2012.08688.x 22747490

[29] Wagner A., Duman R., Henderson K., and Mykhaylyk V. (2016) In-vacuum long-wavelength macromolecular crystallography. Acta Crystallogr. D Struct. Biol. 72, 430–439 10.1107/S2059798316001078 26960130 PMC4784674

[30] Holm L., and Rosenström P. (2010) Dali server: conservation mapping in 3D. Nucleic Acids Res. 38, W545–W549 10.1093/nar/gkq366 20457744 PMC2896194

[31] Ziegler M. O., Jank T., Aktories K., and Schulz G. E. (2008) Conformational changes and reaction of clostridial glycosylating toxins. J. Mol. Biol. 377, 1346–1356 10.1016/j.jmb.2007.12.065 18325534

[32] Coutinho P. M., Deleury E., Davies G. J., and Henrissat B. (2003) An evolving hierarchical family classification for glycosyltransferases. J. Mol. Biol. 328, 307–317 10.1016/S0022-2836(03)00307-3 12691742

[33] Shi W. W., Jiang Y. L., Zhu F., Yang Y. H., Shao Q. Y., Yang H. B., Ren Y. M., Wu H., Chen Y., and Zhou C. Z. (2014) Structure of a novel *O*-linked *N*-acetyl-d-glucosamine (*O*-GlcNAc) transferase, GtfA, reveals insights into the glycosylation of pneumococcal serine-rich repeat adhesins. J. Biol. Chem. 289, 20898–20907 10.1074/jbc.M114.581934 24936067 PMC4110296

[34] Jaffe E. K., and Cohn M. (1978) ^31^P nuclear magnetic resonance spectra of the thiophosphate analogues of adenine nucleotides: effects of pH and Mg^2+^ binding. Biochemistry 17, 652–657 10.1021/bi00597a014 23826

[35] Gout E., Rebeillé F., Douce R., and Bligny R. (2014) Interplay of Mg^2+^, ADP, and ATP in the cytosol and mitochondria: unravelling the role of Mg^2+^ in cell respiration. Proc. Natl. Acad. Sci. U.S.A. 111, E4560–E4567 10.1073/pnas.1406251111 25313036 PMC4217410

[36] Duus J., Gotfredsen C. H., and Bock K. (2000) Carbohydrate structural determination by NMR spectroscopy: modern methods and limitations. Chem. Rev. 100, 4589–4614 10.1021/cr990302n 11749359

[37] Roslund M. U., Tähtinen P., Niemitz M., and Sjöholm R. (2008) Complete assignments of the ^1^H and ^13^C chemical shifts and J(H,H) coupling constants in NMR spectra of d-glucopyranose and all d-glucopyranosyl-d-glucopyranosides. Carbohydr. Res. 343, 101–112 10.1016/j.carres.2007.10.008 17980865

[38] Qasba P. K., Ramakrishnan B., and Boeggeman E. (2005) Substrate-induced conformational changes in glycosyltransferases. Trends Biochem. Sci. 30, 53–62 10.1016/j.tibs.2004.11.005 15653326

[39] Ramakrishnan B., Boeggeman E., Ramasamy V., and Qasba P. K. (2004) Structure and catalytic cycle of beta-1,4-galactosyltransferase. Curr. Opin Struct. Biol. 14, 593–600 10.1016/j.sbi.2004.09.006 15465321

[40] Jank T., Belyi Y., and Aktories K. (2015) Bacterial glycosyltransferase toxins. Cell Microbiol. 17, 1752–1765 10.1111/cmi.12533 26445410

[41] Ardèvol A., Iglesias-Fernández J., Rojas-Cervellera V., and Rovira C. (2016) The reaction mechanism of retaining glycosyltransferases. Biochem. Soc. Trans. 44, 51–60 10.1042/BST20150177 26862188

[42] Albesa-Jové D., and Guerin M. E. (2016) The conformational plasticity of glycosyltransferases. Curr. Opin. Struct. Biol. 40, 23–32 10.1016/j.sbi.2016.07.007 27450114

[43] Vocadlo D. J., Davies G. J., Laine R., and Withers S. G. (2001) Catalysis by hen egg-white lysozyme proceeds via a covalent intermediate. Nature 412, 835–838 10.1038/35090602 11518970

[44] Jamaluddin H., Tumbale P., Withers S. G., Acharya K. R., and Brew K. (2007) Conformational changes induced by binding UDP-2F-galactose to α-1,3 galactosyltransferase: implications for catalysis. J. Mol. Biol. 369, 1270–1281 10.1016/j.jmb.2007.04.012 17493636

[45] Ha S., Walker D., Shi Y., and Walker S. (2000) The 1.9 Å crystal structure of *Escherichia coli* MurG, a membrane-associated glycosyltransferase involved in peptidoglycan biosynthesis. Protein Sci. 9, 1045–1052 10.1110/ps.9.6.1045 10892798 PMC2144650

[46] Berglund H., Olerenshaw D., Sankar A., Federwisch M., McDonald N. Q., and Driscoll P. C. (2000) The three-dimensional solution structure and dynamic properties of the human FADD death domain. J. Mol. Biol. 302, 171–188 10.1006/jmbi.2000.4011 10964568

[47] Esposito D., Sankar A., Morgner N., Robinson C. V., Rittinger K., and Driscoll P. C. (2010) Solution NMR investigation of the CD95/FADD homotypic death domain complex suggests lack of engagement of the CD95 C terminus. Structure 18, 1378–1390 10.1016/j.str.2010.08.006 20947025

[48] Gibson D. G., Young L., Chuang R. Y., Venter J. C., Hutchison C. A. 3rd, and Smith H. O. (2009) Enzymatic assembly of DNA molecules up to several hundred kilobases. Nat. Methods 6, 343–345 10.1038/nmeth.1318 19363495

[49] Ramakrishnan V., Finch J. T., Graziano V., Lee P. L., and Sweet R. M. (1993) Crystal structure of globular domain of histone H5 and its implications for nucleosome binding. Nature 362, 219–223 10.1038/362219a0 8384699

[50] Biasini M., Bienert S., Waterhouse A., Arnold K., Studer G., Schmidt T., Kiefer F., Gallo Cassarino T., Bertoni M., Bordoli L., and Schwede T. (2014) SWISS-MODEL: modelling protein tertiary and quaternary structure using evolutionary information. Nucleic Acids Res. 42, W252–W258 10.1093/nar/gku340 24782522 PMC4086089

[51] Kabsch W. (2010) XDS. Acta Crystallogr. D Biol. Crystallogr. 66, 125–132 10.1107/S0907444909047337 20124692 PMC2815665

[52] Schneider T. R., and Sheldrick G. M. (2002) Substructure solution with SHELXD. Acta Crystallogr. D Biol. Crystallogr. 58, 1772–1779 10.1107/S0907444902011678 12351820

[53] Emsley P., and Cowtan K. (2004) Coot: model-building tools for molecular graphics. Acta Crystallogr. D Biol. Crystallogr. 60, 2126–2132 10.1107/S0907444904019158 15572765

[54] Skubák P., and Pannu N. S. (2013) Automatic protein structure solution from weak X-ray data. Nat. Commun. 4, 2777 24231803 10.1038/ncomms3777 PMC3868232

[55] Vagin A., and Teplyakov A. (2000) An approach to multi-copy search in molecular replacement. Acta Crystallogr. D Biol. Crystallogr. 56, 1622–1624 10.1107/S0907444900013780 11092928

[56] Murshudov G. N., Vagin A. A., and Dodson E. J. (1997) Refinement of macromolecular structures by the maximum-likelihood method. Acta Crystallogr. D Biol. Crystallogr. 53, 240–255 10.1107/S0907444996012255 15299926

[57] Adams P. D., Afonine P. V., Bunkóczi G., Chen V. B., Davis I. W., Echols N., Headd J. J., Hung L. W., Kapral G. J., Grosse-Kunstleve R. W., McCoy A. J., Moriarty N. W., Oeffner R., Read R. J., Richardson D. C., et al (2010) PHENIX: a comprehensive Python-based system for macromolecular structure solution. Acta Crystallogr. D Biol. Crystallogr. 66, 213–221 10.1107/S0907444909052925 20124702 PMC2815670

[58] Pettersen E. F., Goddard T. D., Huang C. C., Couch G. S., Greenblatt D. M., Meng E. C., and Ferrin T. E. (2004) UCSF Chimera: a visualization system for exploratory research and analysis. J. Comput. Chem. 25, 1605–1612 10.1002/jcc.20084 15264254

