# Supplementary material for: Structural basis for the glycosyltransferase activity of the Salmonella effector SseK3
Source: J Biol Chem. 2018 Feb 15;293(14):5064–78. doi: 10.1074/jbc.RA118.001796 (PMC5892559; doi:10.1074/jbc.RA118.001796)
Supplement: Supporting Information [file supp_293_14_5064__index.html]

Structural basis for the glycosyltransferase activity of the Salmonella effector SseK3 — SseK3 is a retaining type-A glycosyltransferase — Structural basis for the glycosyltransferase activity of the Salmonella effector SseK3 — SseK3 is a retaining type-A glycosyltransferase — Supporting Information 

# Structural basis for the glycosyltransferase activity of the *Salmonella* effector SseK3

## Supporting Information

- Supporting Information - PDF of Supporting Information
