# Supplementary material for: Structural basis for the glycosyltransferase activity of the Salmonella effector SseK3
Source: J Biol Chem. 2018 Feb 15;293(14):5064–78. doi: 10.1074/jbc.RA118.001796 (PMC5892559; doi:10.1074/jbc.RA118.001796)
Supplement: Supporting Information [file supp_RA118.001796_135139_1_supp_65978_p3f96k.pdf]

Supporting Information
for

Structural basis for the glycosyltransferase activity of the Salmonella effector SseK3

Diego Esposito, Regina A. Gunster, Luigi Martino, Kamel El Omari, Armin Wagner, Teresa L. M. Thurston, Katrin Rittinger

Figure S1. Binding affinity of UDP-GlcNAc for $SseK3_{14\text{-}335}$. S-2

Figure S2. Structural features of SseK3 and its ligand. S-3

Figure S3. UDP-GlcNAc hydrolysis by NMR. S-4

Figure S4. Arg-GlcNacylation gel assay of SseK3 mutants. S-5

Figure S5. Sequence conservation in the SseK family S-6

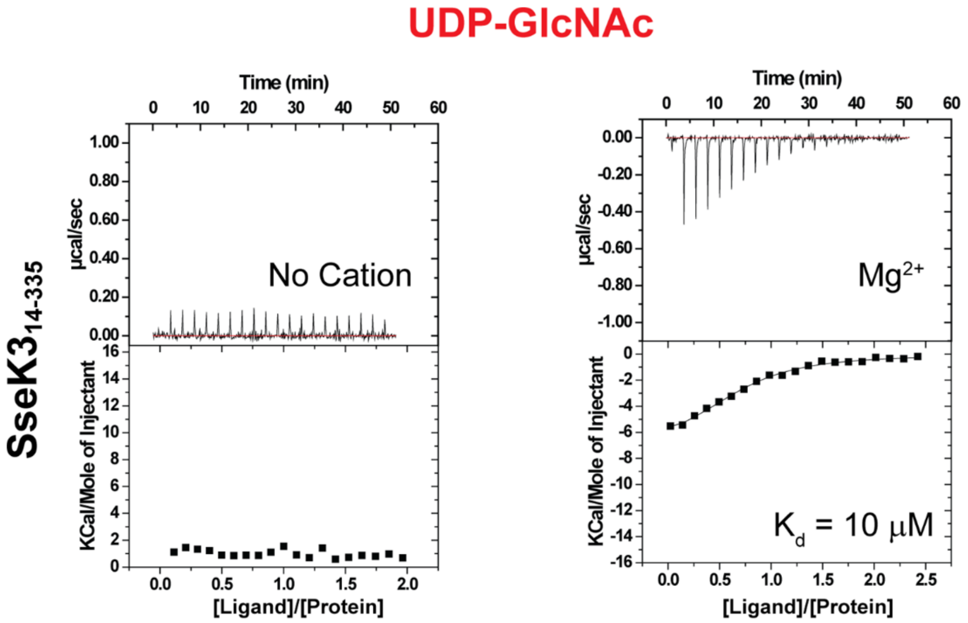

Figure S1. The presence of a divalent cation is necessary for binding of UDP-GlcNac to SseK3. Isothermal Titration Calorimetry curves for the interaction of $SseK3_{14\text{-}335}$ with UDP-Glucosamine (UDP-GlcNAc) in the absence of coordinating divalent cation (left panel) and in the presence of 5 mM $MgCl_2$ (right panel). The integrated heat for the binding interaction was integrated as a function of the molar ratio of titrant to protein in the cell. The data was fitted to a 1:1 binding model and the dissociation constant is reported in each panel.

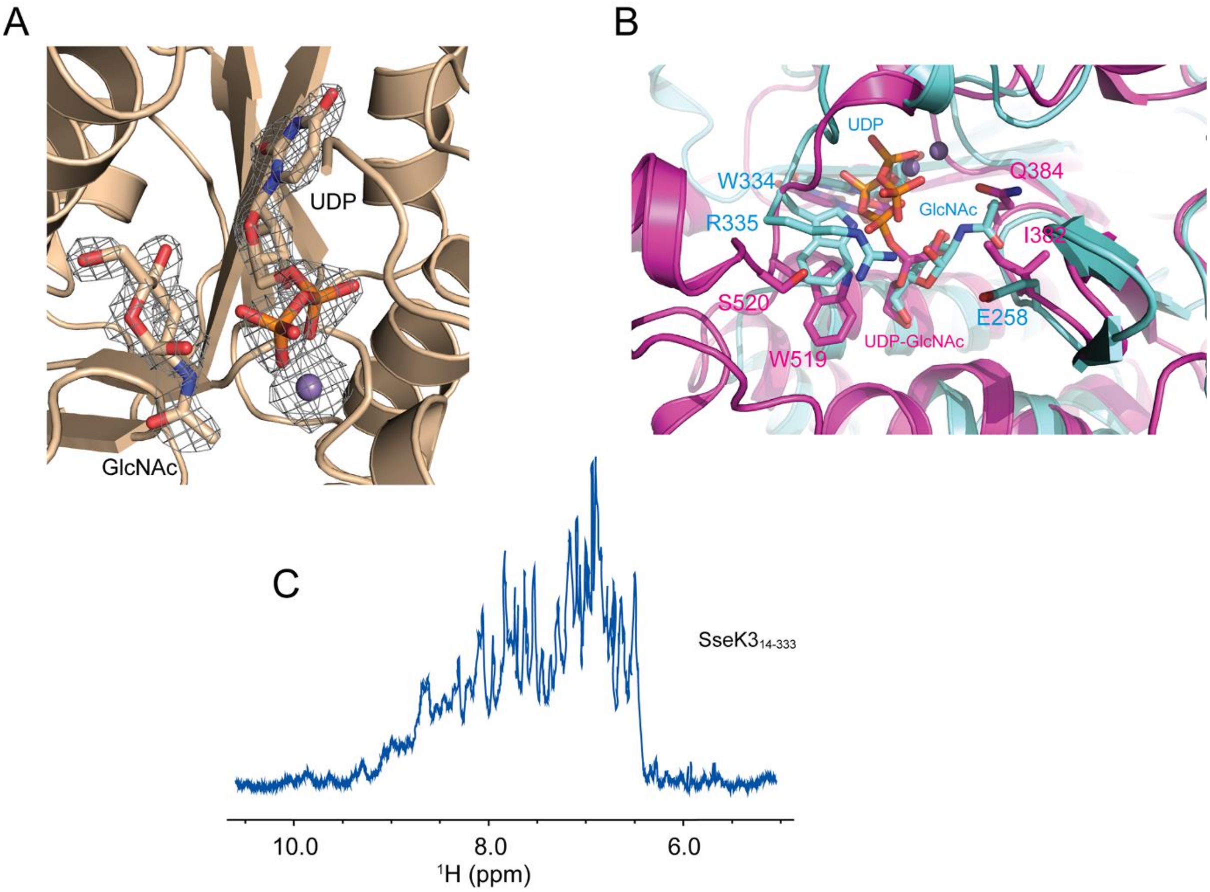

Figure S2. Ligand binding to SseK3. (A) 2Fo-Fc electronic density map around UDP and GlcNAc in the active site pocket of $SseK3_{14\text{-}335}$. The map is plotted at 2σ around the UDP and 1σ around the N-acetyl α-D-glucosamine. (B) Overlap of the structure of the active site of *C. difficile* toxin A (PDB code 3SRZ) with its ligand UDP-Glucose (magenta) and SseK3 with UDP/GlcNAc (cyan). Residues in the different chains are coloured accordingly. (C) Downfield region of the proton NMR spectra of $SseK3_{14\text{-}333}$.

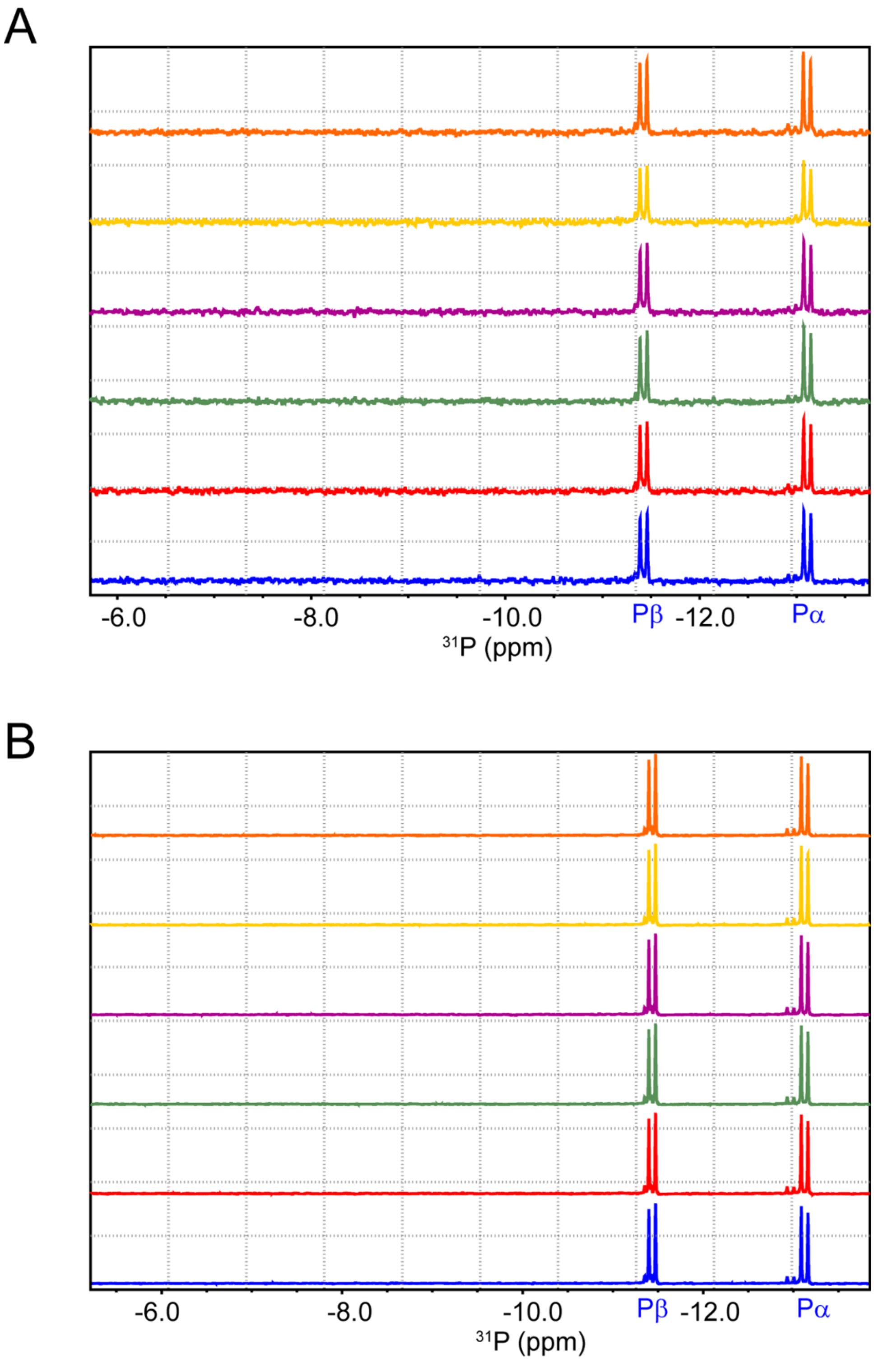

Figure S3. Ligand hydrolysis by SseK3$_{14\text{-}333}$. ^{31}P NMR spectra of a 500 μM solution of UDP-GlcNAC in the presence (A) and absence (B) of 10 μM SseK3$_{14\text{-}333}$. The spectra were recorded at different time points: 0 min (blue), 5 min (red), 15 min (green), 30 min (purple), 90 min (yellow) and 12 hours (orange). The UDP-GlcNAc Pα and Pβ doublets are assigned in the relative spectra.

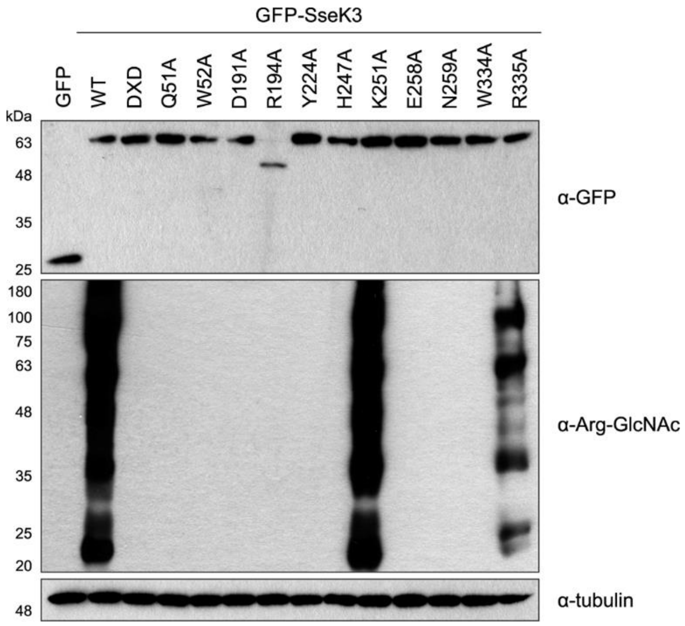

Figure S4. Expression of and Arg-GlcNAcylation induced by putative SseK3 catalytic mutants. 293ET whole cell lysates from Figure 5B were immunoblotted with anti-GFP antibody to test for expression of the GFP-tagged SseK3 variants during NF-κB reporter experiments. Lysates were also blotted with anti-Arg-GlcNAc antibody and anti-tubulin antibody as a loading control. Data shown is representative of 5 independent experiments. DXD corresponds to the SseK3 D226A/D228A mutant.

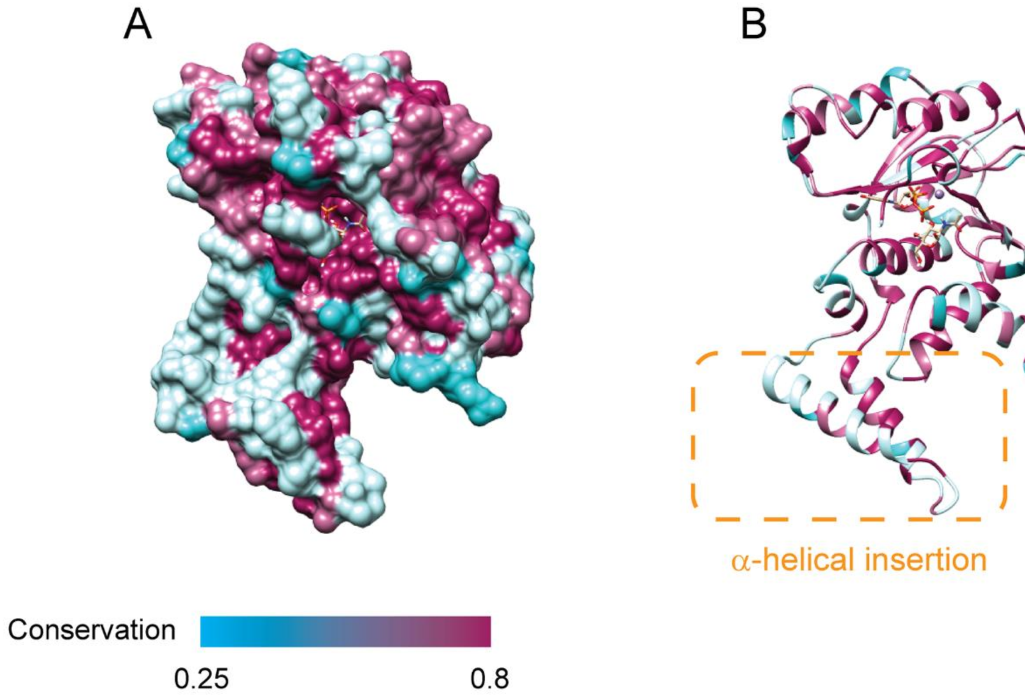

Figure S5. Sequence conservation among the SseK effector family. SseK3 solvent-accessible surface coloured according to sequence conservation among the SseK proteins and their ortholog in *E. coli* NleB. The poorly conserved α-helical insertion is highlighted on the structure of $SseK3_{14-335}$.
